# Public Health Surveillance Systems in the Eastern Mediterranean Region: Bibliometric Analysis of Scientific Literature

**DOI:** 10.2196/32639

**Published:** 2021-11-01

**Authors:** Randa K Saad, Mohannad Al Nsour, Yousef Khader, Magid Al Gunaid

**Affiliations:** 1 Global Health Development|Eastern Mediterranean Public Health Network Amman Jordan; 2 Public Health Faculty of Medicine Jordan University of Science and Technology Irbid Jordan

**Keywords:** public health, surveillance, Eastern Mediterranean Region, bibliometric analysis, literature, research, review

## Abstract

**Background:**

The Eastern Mediterranean Region (EMR) hosts some of the world’s worst humanitarian and health crises. The implementation of health surveillance in this region has faced multiple constraints. New and novel approaches in surveillance are in a constant state of high and immediate demand. Identifying the existing literature on surveillance helps foster an understanding of scientific development and thus potentially supports future development directions.

**Objective:**

This study aims to illustrate the scientific production, quantify the scholarly impact, and highlight the characteristics of publications on public health surveillance in the EMR over the past decade.

**Methods:**

We performed a Scopus search using keywords related to public health surveillance or its disciplines, cross-referenced with EMR countries, from 2011 to July 2021. Data were exported and analyzed using Microsoft Excel and Visualization of Similarities Viewer. Quality of journals was determined using SCImago Journal Rank and CiteScore.

**Results:**

We retrieved 1987 documents, of which 1927 (96.98%) were articles or reviews. There has been an incremental increase in the number of publications (exponential growth, *R^2^*=0.80) over the past decade. Publications were mostly affiliated with Iran (501/1987, 25.21%), the United States (468/1987, 23.55%), Pakistan (243/1987, 12.23%), Egypt (224/1987, 11.27%), and Saudi Arabia (209/1987, 10.52%). However, Iran only had links with 40 other countries (total link strength 164), and the biggest collaborator from the EMR was Egypt, with 67 links (total link strength 402). Within the other EMR countries, only Morocco, Lebanon, and Jordan produced ≥79 publications in the 10-year period. Most publications (1551/1987, 78.06%) were affiliated with EMR universities. Most journals were categorized as medical journals, and the highest number of articles were published in the Eastern Mediterranean Health Journal (SCImago Journal Rank 0.442; CiteScore 1.5). Retrieved documents had an average of 18.4 (SD 125.5) citations per document and an h-index of 66. The top-3 most cited documents were from the Global Burden of Diseases study. We found 70 high-frequency terms, occurring ≥10 times in author keywords, connected in 3 clusters. *COVID-19*, *SARS-CoV-2*, and *pandemic* represented the most recent 2020 cluster.

**Conclusions:**

This is the first research study to quantify the published literature on public health surveillance and its disciplines in the EMR. Research productivity has steadily increased over the past decade, and Iran has been the leading country publishing relevant research. Recurrent recent surveillance themes included COVID-19 and SARS-CoV-2. This study also sheds light on the gaps in surveillance research in the EMR, including inadequate publications on noncommunicable diseases and injury-related surveillance.

## Introduction

### Background

Throughout history, the concepts of population surveillance and public health surveillance (PHS) have been in a continuous state of evolution [1]. In 1968, the 21st World Health Assembly embraced surveillance as a concept and acknowledged its 3 core functions: a systematic collection of data, methodical analysis and evaluation of the data, and timely dissemination of the results, especially to those who can take action [2]. Today, PHS is considered a fundamental function of public health practice and is defined by the World Health Organization (WHO) as the “continuous and systematic collection, orderly consolidation and evaluation of pertinent data with prompt dissemination of results to those who need to know, particularly those who are in a position to take action” [3].

Global commitment to surveillance was recognized in 2005 by the WHO when it revised and adopted the International Health Regulation treaty, defining specific events that require reporting to the WHO within 24 hours of their occurrence [4]. These regulations necessitate that countries have the capacity to detect, assess, report, and respond to public health events; however, only approximately one-third of the world has the capacity to implement this [5]. Technical, political, and economic challenges pose barriers to the implementation of disease surveillance in low- and middle-income countries (LMICs) [6], such as the Eastern Mediterranean Region (EMR) countries.

The EMR comprises 22 member countries, encompassing a total of approximately 679 million individuals [7]. It is host to some of the world’s worst humanitarian and health crises, and >58% of the world’s refugees and internally displaced people come from the EMR [8]. Indeed, the implementation of health surveillance in this region have faced multiple constraints [9], and recent initiatives by the WHO and the US Centers for Disease Control and Prevention (CDC) in collaboration with regional and local networks and governments have been put in place to try to mitigate these constraints and challenges [10,11].

The two main general categories of surveillance include indicator-based surveillance and event-based surveillance [12]. However, new and novel approaches in surveillance are in a constant state of high and immediate demand to directly tackle unexpected health challenges in a timely manner and address community health concerns [13]. A driver of the dynamics of PHS is the broad and diverse cultural, behavioral, economic, and societal differences that affect public health issues in the various countries of the world differently and thus influence the process and implementation of surveillance differently [13]. Four strategies of population surveillance and PHS thus emerge and include passive surveillance, active surveillance, sentinel surveillance, and syndromic surveillance [14]. Other concepts in surveillance have also surfaced, including biosurveillance, which monitors specific data to identify epidemic outbreaks resulting from accidents or bioterrorism [15].

### Objective

Identifying existing literature on a topic helps foster an understanding of the topic’s academic development and thus potentially supports future development directions. This study aims to illustrate the scientific evolution, quantify the scholarly impact, and highlight the characteristics of publications on PHS in the EMR over the last decade. This will provide useful information for the advancement of surveillance strategies to address health issues in the region and will shed light on possible future collaborations and potential joint research engagements.

## Methods

### Search Strategy

We performed our search on June 26, 2021, using the Scopus database, which contains specific useful functions for data mining and bibliometric analysis. Scopus is the largest indexing database; it combines the characteristics of both PubMed and Web of Science, allowing for enhanced utility, both for the literature research and academic needs, including citation analysis [16]. Subject headings as Medical Subject Heading terms or Emtree terms are not directly searchable on Scopus, and instead, Scopus manually assigns index terms (controlled vocabulary) that have a direct relation with the topic [17]. Therefore, in addition to searching abstracts and titles, our search query included the field code KEY, which combines author keywords and indexed terms [17]. To build the query, we used keywords relevant to surveillance and the WHO list of countries in the EMR (Multimedia Appendix 1) [7]. Keywords for surveillance included *population surveillance*, *health surveillance*, *surveillance system*, *biosurveillance*, *passive surveillance*, *active surveillance*, *event-based surveillance*, *indicator-based surveillance,* *case-based surveillance*, *sentinel surveillance*, *syndromic surveillance*, *disease surveillance*, *environmental surveillance*, and *epidemiological surveillance*. We used truncations and wild cards, as appropriate, to maximize the capture of relevant citations. To analyze the most recent publications in the field, which may have implications for future trends in research, we targeted the search to the period from 2010 to 2021. We included papers, reviews, letters, conference papers, and book chapters, as these citations usually report original scientific outputs. No language restrictions were applied, and we included both published papers and papers in the press.

### Scientific Literature Bibliometric Indicators

Data from the retrieved documents were exported to Microsoft Excel. The exported data were used to calculate the following indicators: number of documents published by document type; number of documents published per language; number of documents published per year, and we excluded documents published in 2021 from the analysis of annual trends as this study was conducted in June 2021; number of documents published per journal; number of documents published per country; number of documents published per organizational affiliation, and we combined affiliations into 5 categories—universities in the EMR, universities outside the EMR, Ministries of Health in the EMR, research institutes in the EMR, research institutes outside, and global health institutes; number of documents per funding source; and number of documents published by subject category. We used the subject categories as indexed by Scopus.

### Citations and Quality Assessment

We used the citation overview function on Scopus to determine the mean, median, and range of citations of all retrieved documents, including their h-index. We scrutinized the topmost cited documents, as well as the authors who published >20 papers on the topic.

We assessed the quality of the most productive 20 journals in the field of surveillance in the EMR using the SCImago Journal Rank (SJR) and CiteScore (CS). SJR is a metric that is based on centrality concepts and data from Scopus, and we chose it as it limits self-citations and thus limits falsely inflated quality ranks [18]. We additionally used CS, as it has become the new standard that gives a more comprehensive, transparent, and current view of a journal’s impact [18]. To determine the subject area of journals, we used the subject area and category provided by the SCImago ranking website in addition to reviewing the official scope of each journal on its respective website.

### Visualization of Similarities

Citation information, bibliographical information, abstracts and keywords, and included references were also imported to the Visualization of Similarities Viewer (VOSviewer) software v.1.6.16 (Centre for Science and Technology Studies, Leiden University) to analyze and visualize relationships among authors, countries, and the terms used in the papers [19]. This mapping method was used to estimate the association strength between items, which is indicative of the similarity between terms. The co-occurrence of 2 items in a larger number of documents indicates that the items are very similar to one another. Graphically, each cluster of items (eg, a group of linked keywords) is identified by a different color [20]. We used a resolution of clustering of 2 and a minimum cluster size of 12 to eliminate small clusters. Visualization maps were based on document weights, unless otherwise stated, and the diameter or the label size of an item denotes the number of occurrences in the documents, and the distance between 2 items represents the degree to which they are associated [20]. Network coauthorship analysis was first performed based on the full counting method on VOSviewer, with the unit of analysis being the author. Papers including >25 authors were excluded from this analysis, and both the minimum number of papers published by an author and the minimum number of citations of an author was set at 5 to identify prominent authors who have published on the topic. We also performed the same coauthorship analysis, with the unit being countries. Papers including >25 countries were excluded, and only countries with ≥5 published papers were included. No restriction was placed on the number of citations. Within the coauthorship network, the links attribute indicates the number of coauthorship links of a given country with other countries, whereas the total link strength attribute indicates the total strength of the coauthorship links of a given country with other countries. These numbers are readily provided by VOSviewer in the visualized network.

We used co-occurrence analysis to identify hot topics and time trends of the themes. We used author keywords, occurring >10 times, to identify network associations in underlying research themes among documents on surveillance in the EMR. To maintain the focus on scientific themes and keywords, we omitted the names of countries and regions from the resultant list. We also applied normalization to the analysis based on the association strength to eliminate redundancy in similar keywords that define the same concept. We further constructed an overlay visualization of this relationship to determine the evolution of themes with time during our study period.

## Results

### Volume and Type of Publications

We retrieved 1987 documents, of which 1239 (62.36%) were open access, and only 8 (0.4%) were still in press, whereas all others were in the final published stage. Most (1927/1987, 96.98%) of the retrieved documents were articles or reviews. Conference proceedings and book chapters constituted only 0.65% (13/1987) and 2.37% (47/1987), respectively. Documents were mostly published in English (1930/1987, 97.13%), whereas a minority were published in Persian, French, Arabic, Turkish, Spanish, Italian, or Chinese.

### Annual Trend of Publications

**Figure 1:**
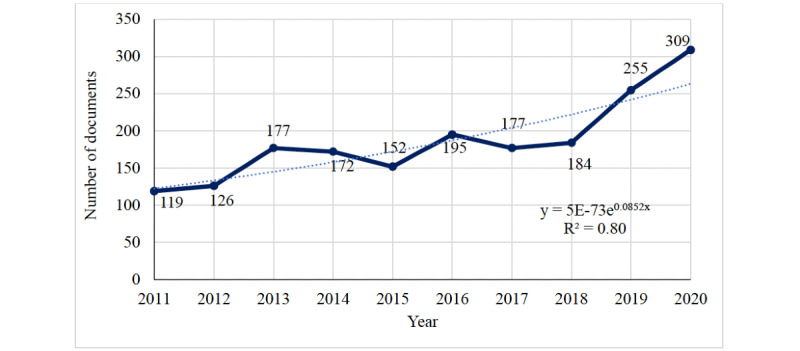
Number of published documents on surveillance in the Eastern Mediterranean region between 2011 and 2020. Dotted line represents exponential growth with R^2^=0.8.

Between 2011 and 2020, there was an incremental increase in the number of scientific publications, corresponding to an exponential growth model (*R^2^*=0.80; Figure 1). Publications in 2020 were 2.6 times greater in number than those published in 2011. During the entire 10-year period, there was a surge of publications seen in 2013-2014, 2016, 2019, and 2020. A review of papers published during these periods showed a high percentage (>10%) of publications on the following: novel Middle East respiratory syndrome coronavirus in 2013-2014 (44/1987, 2.21% of documents; 46/349, 13.2% of the documents published in 2013-2014), influenza in 2016 (32/1987, 1.61% of documents; 32/195, 16.4% of the documents published in 2016) and 2019 (29/1987, 1.46% of documents; 28/255, 10.9% of the documents published in 2019), and COVID-19 in 2020 (59/1987, 2.97% of documents; 60/314, 19.1% of the documents published in 2020).

### Publication Distribution by Country

Most publications on surveillance in the EMR were publications affiliated with Iran (501/1987, 25.21%) and the United States (468/1987, 23.55%), followed by Pakistan (243/1987, 12.23%), Egypt (224/1987, 11.27%), and Saudi Arabia (209/1987, 10.52%; Figure S1A in Multimedia Appendix 2). Among the other EMR countries, only Morocco, Lebanon, and Jordan produced ≥79 publications (≥4%) in the 10-year period, whereas 11 countries produced ≤50 publications, particularly Somalia, Syria, Bahrain, and Libya, each contributing <1% (n=4-17) to the publication output (Figure S1B in Multimedia Appendix 2).

### Publication Distribution by Affiliation

Each publication had one or more institutional affiliation. Most of the published documents (1562/1987, 78.61%) were affiliated with universities in the EMR, whereas some (745/1987, 37.49%) were affiliated with universities from outside the region. Ministries of health, including those of Egypt, Iraq, Sudan, Kuwait, Qatar, Oman, Lebanon, Saudi Arabia, and Iran, contributed to approximately 17.41% (346/1987) of the published literature, with the Iranian Ministry of Health and Medical Education being the major contributor. Similarly, global health institutes, including the CDC, WHO, National Health Institute, and the Global Health Development/Eastern Mediterranean Public Health Network (GHD/EMPHNET), European Centre for Disease Prevention and Control, National Center for Emerging and Zoonotic Infectious Diseases, Medecins Sans Frontieres, and Food and Agriculture Organization of the United Nations, contributed to approximately 17.41% (346/1987) of the published literature. The top 5 most productive universities in the region were all in Iran, and included: Tehran University of Medical Sciences, Shahid Beheshti University of Medical Sciences, Aga Khan University, Iran University of Medical Sciences, and Shiraz University of Medical Sciences. The top 5 most productive universities outside the region were Johns Hopkins University, Imperial College London, London School of Hygiene and Tropical Medicine, University of Oxford, and The University of Sydney. As for the most productive research institutes, the top 5 in the EMR were the Endocrinology and Metabolism Research Institute in Iran, Pasteur Institute of Iran, National Institute of Health Pakistan, National Research Centre in Egypt, and the Noncommunicable Diseases Research Center in Iran; and Public Health England, Paris Institut Pasteur, Karolinska Institutet, Institut national de la santé et de la recherche médicale, and the International Centre for Diarrhoeal Disease Research Bangladesh, were the top 5 outside the EMR (Multimedia Appendix 2 Figure S2).

### Source of Funding

Most of the identified publications did not report a source of funding (1415/1987, 71.21%); however, for those that did, most were funded by the US Department of Health and Human Services (121/572, 21.2%) or by the National Institute of Health (83/572, 14.5%). Department of Health and Human Services and National Institute of Health–funded publications substantially peaked in 2015 and in 2019, with an obvious decline in 2020, and these funded studies were mainly published by the United States. The CDC, European Commission, Bill and Miranda Gates Foundation, and the WHO funded approximately 38 to 42 papers each.

### Most Productive Citing Journals

Of the 1987 published literature, 1931 (97.18%) documents were published in 160 distinct scientific journals, whereas the rest were included in conference proceedings, books, or their sources were not defined. Table 1 lists the top 20 most productive journals publishing the greatest number of papers related to the field of surveillance in the EMR between 2011 and 2021. Their median 2020 SJR was 1.123 (range 0.182-3.44), and the Eastern Mediterranean Health Journal (SJR 0.442, CS 1.5) topped the list, publishing approximately 3.82% (76/1987) of the literature retrieved on the topic. Most journals were categorized as medical journals, and on reviewing the scope of each of the top 20 journals, only 2 journals, Eurosurveillance [21] and JMIR Public Health and Surveillance [22], specifically focused on surveillance.

**Table 1:** The SCImago Journal Rank (SJR), CiteScore (CS), and h-index of the 20 most productive journals in the field of public health surveillance in the Eastern Mediterranean Region between 2011 and 2021, arranged by productivity (N=1931).

Top 20 journals	2020 SJR	CS	h-index	Values, n (%)^a^	Journal subject area and category
Eastern Mediterranean Health Journal	0.442	1.5	47	76 (3.93)	Medicine (miscellaneous)
PLOS One	0.990	5.3	332	61 (3.16)	Multidisciplinary (sciences)
International Journal of Infectious Diseases	1.278	7.0	89	45 (2.33)	Medicine (miscellaneous, ID^b^, and medical microbiology)
PLOS Neglected Tropical Diseases	1.990	7.1	135	42 (2.18)	Medicine (ID and PHEOH^c^), pharmacology, toxicology, and pharmaceutics
Emerging Infectious Diseases	2.540	9.8	226	37 (1.92)	Medicine (epidemiology, ID, and medical microbiology)
Journal of Infection and Public Health	0.983	4.9	35	35 (1.81)	Medicine (miscellaneous, ID and PHEOH)
Journal of Infection in Developing Countries	0.322	1.6	49	29 (1.5)	Medicine (miscellaneous and ID) and immunology and microbiology
Vaccine	1.585	5.6	184	28 (1.45)	Medicine (ID and PHEOH), immunology and microbiology, veterinary (miscellaneous), biochemistry, and genetics and molecular biology (molecular medicine)
Journal of Infectious Diseases	2.690	9.2	252	27 (1.4)	Medicine (immunology and allergy and ID)
Archives of Iranian Medicine	0.490	2.3	47	26 (1.35)	Medicine (miscellaneous)
Eurosurveillance	2.766	13.9	104	26 (1.35)	Medicine (epidemiology, ID, and PHEOH) and immunology and microbiology (virology)
Iranian Journal of Epidemiology	0.182	0.7	11	26 (1.35)	Medicine (epidemiology)
BMC^d^ Infectious Diseases	1.278	4.4	104	22 (1.14)	Medicine (ID)
Iranian Journal of Public Health	0.452	2.1	39	22 (1.14)	Medicine (PHEOH)
Clinical Infectious Diseases	3.440	13.2	336	21 (1.09)	Medicine (ID and medical microbiology)
Acta Tropica	0.969	5.2	101	18 (0.93)	Medicine (ID), immunology and microbiology (parasitology), veterinary (miscellaneous), and agricultural and biological sciences (insect sciences)
Transboundary and Emerging Diseases	1.392	7.6	63	18 (0.93)	Medicine (miscellaneous), immunology and microbiology (miscellaneous), and veterinary (miscellaneous)
American Journal of Tropical Medicine and Hygiene	1.015	4.0	151	16 (0.83)	Medicine (miscellaneous and ID) and immunology and microbiology (parasitology and virology)
BMC Public Health	1.230	4.1	143	16 (0.83)	Medicine (PHEOH)
Epidemiology and Infection	0.992	5.0	109	16 (0.83)	Medicine (epidemiology and ID)
JMIR Public Health and Surveillance	1.446	5.8	146	15 (0.78)	Not available on SCImago, description from journal website [22]: public health and technology, public health informatics, mass media campaigns, surveillance, participatory epidemiology, and innovation in public health practice and research
Journal of Medical Virology	0.782	11.6	121	14 (0.73)	Medicine (ID) and immunology and microbiology (virology)

^a^The total number of documents retrieved that were published in journals was 1931. Percentages were calculated as the (number of documents published by each journal × 100) / 1931.

^b^ID: infectious diseases.

^c^PHEOH: Public Health, Environmental, and Occupational Health.

^d^BMC: BioMed Central.

### Publications by Subject Category

Each published document was categorized under one or more subject categories in Scopus. Most of the retrieved literature (1622/1987, 81.63%) were categorized under medicine as a subject. Approximately 20.43% (406/1987) were categorized under immunology and microbiology. Of those categorized under medicine, 90.14% (1462/1622) were labeled with indexed keywords related to surveillance: disease surveillance, population surveillance, surveillance, PHS, or sentinel surveillance.

### Citation Metrics

The retrieved documents received a total of 36,630 citations, with an average of 18.4 (SD 125.5) citations per document (median 5, range 0-4402) and an h-index of 66. The number of documents receiving ≥10, ≥50, or ≥100 citations was 679, 100, and 41 documents, respectively. The top-3 most cited documents were all from the ongoing Global Burden of Diseases (GBD) study and were open access papers published in The Lancet by the US Institute for Health Metrics and Evaluation, assessing global, national, and regional all-cause and specific-cause mortality [23-25]. These 3 global studies, which were published between 2015 and 2018, had >700-1000 collaborators or authors each and were cited 1729-4409 times [23-25]. Papers from the GBD study comprise 47% (7/15) of the most cited papers on the topic of surveillance in the EMR. The other most cited papers were also open-access global or multicounty studies, 2 of which were conducted by the CDC in collaboration with the WHO and assessed rotavirus infections or vaccines [26,27], and 2 were reviews assessing hepatocellular carcinoma [28] or hepatitis C virus [29], and all recommended the implementation of surveillance systems. The 6 authors, including 1 female author, who published >20 documents on the topic (range 20-42), were from Iran, Saudi Arabia, and Jordan. Their average h-index on Scopus was 55 (SD 32; median 51, range 18-98), and their median citation frequency was 17,236 (range 5198-85,491).

### Visualization of Similarities and Associations

#### Coauthorship

**Figure 2:**
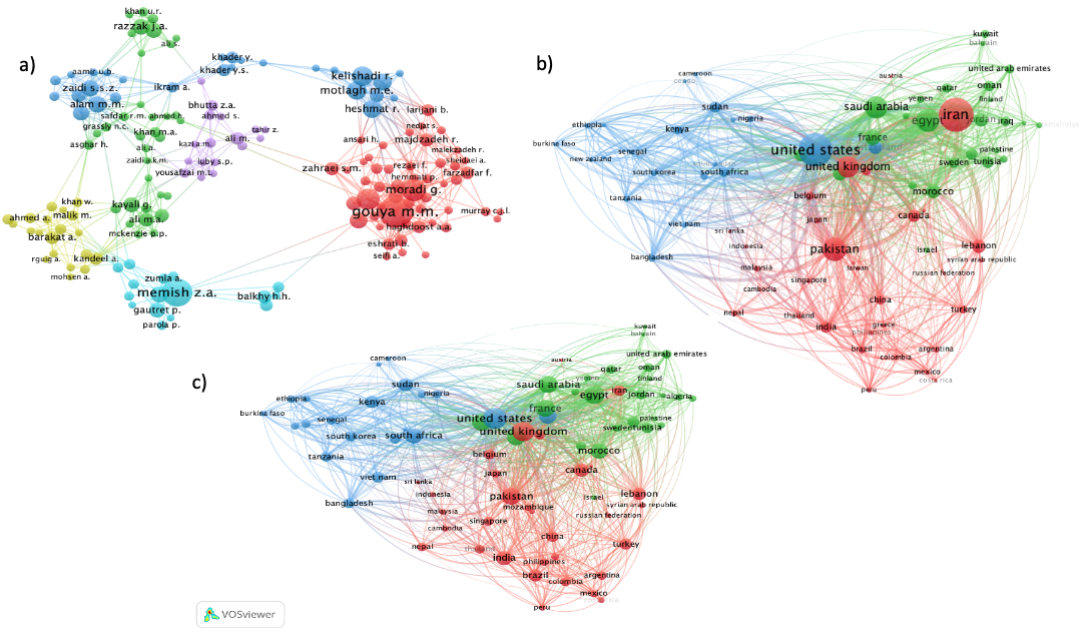
(A) Visualization of Similarities Viewer (VOSviewer) network of author coauthorship map representing 6 clusters of collaborations on surveillance research in the Eastern Mediterranean region, 2011-2021. Included authors (N=165) were those with at least 5 publications, with <25 authors per publication, and have been cited at least 5 times. (B) VOSviewer network of country coauthorship map weighted by the number of documents, representing 5 clusters of collaborations on surveillance research in the Eastern Mediterranean region, 2011-2021. Included countries (N=78) were those with at least 5 publications, with <25 countries collaborating per publication. (C) VOSviewer network of country coauthorship map, weighted by number of links, representing 5 clusters of collaborations on surveillance research in the Eastern Mediterranean region, 2011-2021. Included countries (N=78) were those with at least 5 publications, with <25 countries collaborating per publication. VOSviewer: Visualization of Similarities Viewer.

In papers with <25 authors, 173 authors had at least 5 publications and were cited at least 5 times. Of these 173 authors, 165 (95.4%) authors were connected (have collaborated) in 6 distinct clusters (Figure 2A). Similarly, excluding papers coauthored by ≥25 countries, 78 countries had ≥5 papers published on the topic of surveillance in the EMR, and these countries were connected in 5 different coauthorship clusters (Figure 2B). In these figures, the size of circles represents the number of documents published by the author or country, and the thickness of the lines depicts the size of the collaboration between the authors or countries (Figures 2A and 2B, respectively). The EMR country publishing the most on the topic was Iran; however, it lags behind other countries in terms of collaboration and only has links with 40 other countries, with a total link strength of 164. The biggest collaborator from the EMR countries was Egypt, with 67 links and a total link strength of 402, followed by Pakistan, with 62 links and a total link strength of 390; Saudi Arabia, with 62 links and a total link strength of 307; Morocco, with 55 links and a total link strength of 206; and Lebanon, with 46 links and a total link strength of 177. The United States is the country outside the EMR with the most collaboration links (n=76), with a link strength of 890 (Figure 2C).

#### Co-occurrence

**Figure 3:**
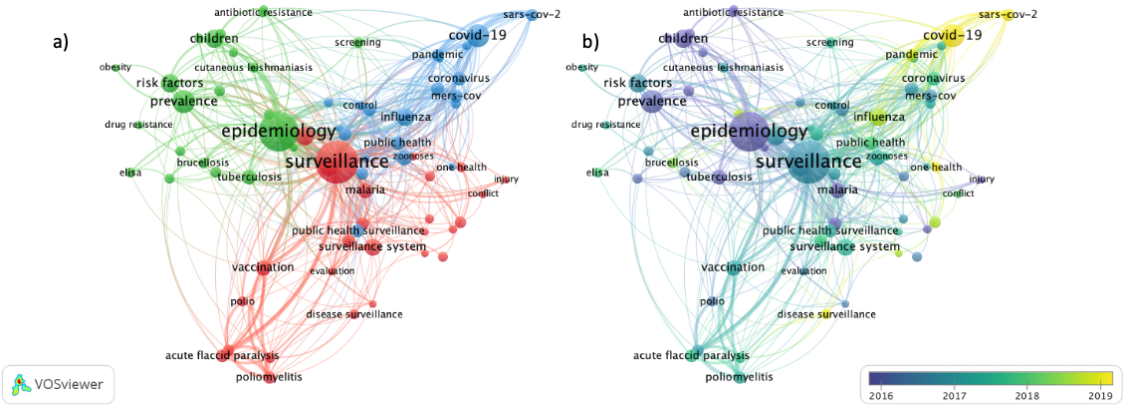
(A) Visualization of Similarities Viewer (VOSviewer) network of author keyword co-occurrence map weighted by occurrence, representing three clusters of keywords relating to surveillance research in the Eastern Mediterranean Region, 2011-2021. Included keywords (N=70) were those occurring at least 10 times. (B) VOSviewer overlay visualization by time of author keyword relating to surveillance research in the Eastern Mediterranean Region, 2011-2021, weighted by occurrence and scored by the average publications per year. Included keywords (N=70) were those occurring at least 10 times. VOSviewer: Visualization of Similarities Viewer.

A knowledge map of author keyword co-occurrence is shown in Figure 3A. We found 70 high-frequency terms occurring at least 10 times in the author keywords. These keywords were connected in 3 distinct clusters. As expected, COVID-19, SARS-CoV-2, and pandemic represented the most recent 2020 cluster (Figure 3B).

## Discussion

### Principal Findings

To the best of our knowledge, this is the first bibliometric study to assess publications on PHS and its disciplines during the last decade in the EMR. The information presented in this study shows the growth of publications between 2011 and 2021. It quantifies the contributions of countries, journals, organizations, and authors to this field and illustrates the collaborative behaviors between authors and countries. It also analyzes the documents by subject category and maps out the co-occurrence and time trends of relevant keywords and terms.

There was an exponential increase in publications on this topic in the EMR in the past decade, with a 2.6-fold increment in published papers in 2020 compared with 2011. This evolution of publications in the EMR mirrors the global trends of interest in health surveillance [30]. The peaks in the number of documents published in the 2013-2014 period and the years 2016 and 2020 might have been driven by certain disease outbreaks or the launch of pivotal surveillance networks in the region. Indeed, the first case of Middle East respiratory syndrome coronavirus was detected in 2012 in Saudi Arabia [31], and shortly after, multiple studies were published on the topic, contributing to approximately 13.2% (46/349) of publications seen in the following 2 years (2013-2014). Similarly, in 2016, there was a surge in influenza-related publications, which coincides with the official launch of the Eastern Mediterranean Flu Network, which is a web-based surveillance effort supervised by the WHO Regional Office for the Eastern Mediterranean to strengthen influenza surveillance systems in the region [32]. With the increasing burden of noncommunicable diseases (NCDs) both worldwide and in the region, there has also been an increasing interest in NCD research [33], which might explain the peak in publications in recent years. As for the year 2020, the explosion in publications echoes the scientific frenzy witnessed with the commence of the global COVID-19 pandemic, which took the lead and contributed massively to the literature at both the global and regional levels [34,35].

Papers cited >100 times are often referred to as citation *classics* [36]. Most *classic* papers identified during this search, based on citation counts, were open access papers of multicountry or global studies. The top-cited papers were mostly from the ongoing GBD study, which is the most descriptive worldwide epidemiological study examining the trends of 204 countries, dating back to 1990 [37]. It is a global collaborative initiative, with multiple resultant publications, following the guidelines for accurate and transparent health estimate reporting [37]. Some resultant papers were authored by EMR collaborators, specifically addressing health issues related to the EMR [38], including cardiovascular disease [39], neurodegenerative diseases [40], obesity [41], and child and adolescent injury [42].

Within countries in the region, research production was concentrated in Iran, Egypt, Pakistan, and Saudi Arabia. This is not surprising, as Iran, Egypt, and Pakistan are the most populated countries in the region, with a 2019 population estimate >82, >100, and >216 million individuals, respectively [43]. Similarly, although Saudi Arabia ranks eighth in terms of the total population compared with the other EMR countries, its gross domestic product exceeds 792 billion (2019 World Bank) [43]. For countries contributing to <1% of the retrieved literature, the long-standing devastating state of economic and political turmoil in Somalia [44], Libya [45], and Syria [46] might be the culprit behind these countries’ lack of meaningful contribution to research production on the topic. Bahrain’s low contribution to published research might reflect its small population, given that it is the least populated country in the EMR [43].

Research collaboration between countries does not necessarily mirror the volume of research produced by each country. Although Iran is the most productive country in terms of the volume of publications, its level of coauthorship collaboration lags behind that of Egypt, Pakistan, Saudi Arabia, Morocco, and Lebanon. This low level of scientific collaboration has been linked to the international political and economic sanctions placed on Iran [47,48]. International research collaboration is led by the United States, and the link strength of US collaboration on surveillance research with other countries reflects the concentration of funding agencies in the United States, as most funding, when provided, originated from US-based institutes.

As for organizational affiliations, in addition to connections with universities both inside and outside the region, there is considerable research affiliated with regional ministries of health as well as with global institutes, including the CDC and GHD/EMPHNET. In more than half of the world, health surveillance is carried out by competency-based field epidemiology training programs (FETPs) [49], which are key activities of the CDC in advancing health globally [50]. These programs, which mostly function within ministries of health, have conducted most of the surveillance of emerging infections worldwide and have trained most of the public health workers who manage surveillance systems at a country or regional level [49]. In a recent evaluation study, it was shown that approximately two-thirds of FETP graduates in the EMR are engaged in managing PHS systems or analyzing surveillance data [51]. During the COVID-19 outbreak, the efforts of FETP graduates in supporting the surveillance functions within their countries were witnessed firsthand within the EMR [11]. In this region, FETP programs have been launched and are maintained with the aid of GHD/EMPHNET, which works in close collaboration with the CDC and ministries of health across the region [52].

Very few journals focus on surveillance as a scholarly topic of its own. Instead, surveillance is embedded in medical or multidisciplinary journals, focusing on infectious diseases, immunology and microbiology, or medicine and public health in general. This is not specific to the EMR but is a phenomenon that is seen at the global level [30] and iterates how the current state of surveillance systems depends mostly on disease-specific approaches, limiting its generalizability and effectiveness as a multidisciplinary approach to public health [53].

Within the past few years, the topic of surveillance in the EMR has evolved from the concept of surveillance in general toward the concept of surveillance systems and disease surveillance. However, looking at the keyword co-occurrence map, one cannot help but notice the near lack of terms associated with injury surveillance and NCD surveillance, other than obesity. This is especially alarming given that two-thirds of injury-related deaths occur in LMICs [54], and in the EMR, injury-related mortality and disability are on the rise (both accident and war-related injury) [55]. Almost 19% of the global child-related injury deaths occur in the EMR; however, only a limited number of EMR countries have existing injury surveillance systems or trauma registries [42]. Similarly, the EMR carries a disproportionate burden of NCDs, and it is a region where the delivery of effective NCD interventions remains an overwhelming challenge to health systems [56,57]. The low number of publications on NCD surveillance has been partly attributed to the weak surveillance structure and capacity of these LMICs [58]. Several LMICs are still wrestling with the high prevalence of communicable diseases, and their overburdened health care systems have little capacity, if any, to focus on NCDs [58]. Unlike other NCDs and NCD risk factors, obesity does appear as a keyword on the co-occurrence map, although its co-occurrence is shy from being too significant. During the past 2 decades, with increasing obesity trends in the region, interest in obesity in the EMR has increased [41,59]. This is especially true, given that the rates of obesity among children in the EMR exceed those seen globally [41].

With regard to communicable diseases, several diseases are present within the surveillance cluster on the network map, including measles, malaria, typhoid, polio or poliomyelitis, dengue, and zoonoses. The prominence of the link between surveillance and polio, poliomyelitis, or acute flaccid paralysis, within this cluster, is not surprising, as although wild poliovirus has been mostly eradicated, it remains endemic to 2 countries in the EMR, Afghanistan and Pakistan [60]. Circulating vaccine-derived poliovirus outbreaks have also been reported in Syria and Somalia [61], and recently, new outbreaks have been reported in Yemen and Sudan [62]. Multiple surveillance capacity–building initiatives have been implemented by international health networks, such as the Global Polio Eradication Initiative, spearheaded by the WHO, CDC, the United Nations Children’s Fund, in collaboration with other organizations [62], and by regional public health networks, including the Polio and Routine Immunization Program, spearheaded by GHD/EMPHNET, in collaboration with international agencies and ministries of health across the region [63].

Our study used mature analysis tools and validated methods using Scopus and VOSviewer to determine frequencies, construct trends, and determine associations. The limitations of this study are mostly those inherent to its bibliometric design, including the fact that the number of citations does not necessitate the quality of the publications, and citations in themselves may be misleading, especially when time is factored out of the equation, as citations continue to accumulate over time. Although most papers were retrieved from health-related disciplines, variations between subdisciplines might also artificially skew the results. Similarly, within the EMR, a number of papers are published in local journals; these are not indexed by Scopus and thus are not included in this analysis.

### Conclusions

Our study is the first to quantify the published scholarly literature on health surveillance and its corresponding disciplines in the EMR. It provides an analysis of the scientific research on health surveillance in the EMR, with evidence-based descriptions and visualizations of research output. Research productivity, as measured by the number of publications on the topic, has steadily increased over the past decade. In addition to highlighting collaborations and recurrent surveillance themes, this study identifies leading countries and organizational affiliations publishing PHS-related research. It further describes the patterns of performance and impact of research and sheds light on the gaps in surveillance research in the EMR, including inadequate publications on NCDs and injury-related surveillance.

## Appendix

Multimedia Appendix 1 Scopus search strategy.

Multimedia Appendix 2 Number of published documents in the field of public health surveillance in the Eastern Mediterranean Region (EMR), between 2011 and 2021, (a) by country worldwide and (b) by countries in the EMR. In addition to, affiliations of the published literature in the field of public health surveillance in the EMR 2011 and 2021.

## Abbreviations

CDC: Centers for Disease Control and Prevention
CS: CiteScore
EMR: Eastern Mediterranean Region
FETP: field epidemiology training program
GBD: Global Burden of Diseases
GHD/EMPHNET: Global Health Development/Eastern Mediterranean Public Health Network
LMIC: low- and middle-income country
NCD: noncommunicable disease
PHS: public health surveillance
SJR: SCImago Journal Rank
VOSviewer: Visualization of Similarities Viewer
WHO: World Health Organization
