# Supplementary material for: Public Health Surveillance Systems in the Eastern Mediterranean Region: Bibliometric Analysis of Scientific Literature
Source: JMIR Public Health Surveill. 2021 Nov 1;7(11):e32639. doi: 10.2196/32639 (PMC8593796; doi:10.2196/32639)
Supplement: Multimedia Appendix 1 [file publichealth_v7i11e32639_app1.docx]

## Appendix 1: Scopus Search Strategy

**Concept 1: Eastern Mediterranean Regio Countries**

#1 ( ( ( TITLE-ABS-KEY ( afghani* )  OR  TITLE-ABS-KEY ( bahrain* )  OR  TITLE-ABS-KEY ( egypt* )  OR  TITLE-ABS-KEY ( iran* )  OR  TITLE-ABS-KEY ( iraq* )  OR  TITLE-ABS-KEY ( jordan* )  OR  TITLE-ABS-KEY ( kuwait* )  OR  TITLE-ABS-KEY (leban* ) OR TITLE-ABS-KEY ( libya* )  OR  TITLE-ABS-KEY ( morocc* )  OR  TITLE-ABS-KEY (oman*) OR  TITLE-ABS-KEY ( pakistan* )  OR  TITLE-ABS-KEY ( palestin* )  OR  TITLE-ABS-KEY ( qatar* )  OR  TITLE-ABS-KEY ( saudi )  OR  TITLE-ABS-KEY (ksa) OR TITLE-ABS-KEY ( somali* )  OR  TITLE-ABS-KEY ( sudan* )  OR  TITLE-ABS-KEY ( syria*) OR TITLE-ABS-KEY ( tunisia* )  OR  TITLE-ABS-KEY ( emirat* )  OR  TITLE-ABS-KEY (uae) OR  TITLE-ABS-KEY ( yemen* )  OR  TITLE-ABS-KEY (eastern  PRE/0 mediterranean PRE/0 region) OR  TITLE-ABS-KEY ( middle  PRE/0  east*  PRE/0  region )  OR  TITLE-ABS-KEY ( mena  PRE/0  region )  OR  TITLE-ABS-KEY ( north  PRE/0  africa* ) ) ) )

**Concept 2: Health Surveillance**

#2 ( TITLE-ABS-KEY ( health  PRE/2  surveillance )  OR  TITLE-ABS-KEY ( {Surveillance system} )  OR  TITLE-ABS-KEY ( {biosurveillance} )  OR  TITLE-ABS-KEY ( {bio- surveillance} )  OR  TITLE-ABS-KEY ( {passive surveillance} )  OR  TITLE-ABS-KEY ( {active surveillance} )  OR  TITLE-ABS-KEY ( {sentinel surveillance} )  OR  TITLE-ABS-KEY ( {event-based surveillance} )  OR  TITLE-ABS-KEY ( {event based surveillance}) OR TITLE-ABS-KEY ( {indicator-based surveillance} )  OR  TITLE-ABS-KEY ( {indicator based surveillance} )  OR  TITLE-ABS-KEY ( {case-based surveillance} )  OR  TITLE-ABS-KEY ( {case based surveillance} )  OR  TITLE-ABS-KEY ( {syndromic surveillance} )  OR  TITLE-ABS-KEY ( {disease surveillance} )  OR  TITLE-ABS-KEY ( {population* surveillance}) OR  TITLE-ABS-KEY ( {environment* surveillance} )  OR  TITLE-ABS-KEY ( {epidemiolog* surveillance} ) )

**Limits: Type of Document**

#3 ( LIMIT-TO ( DOCTYPE ,  "ar" )  OR  LIMIT-TO ( DOCTYPE ,  "re" )  OR  LIMIT-TO ( DOCTYPE ,  "le" )  OR  LIMIT-TO ( DOCTYPE ,  "cp" )  OR  LIMIT-TO ( DOCTYPE ,  "ch" )  OR  EXCLUDE ( DOCTYPE ,  "le" ) )

**Limits: Years 2011-2021**

#4 ( LIMIT-TO ( PUBYEAR ,  2021 )  OR  LIMIT-TO ( PUBYEAR ,  2020 )  OR  LIMIT-TO ( PUBYEAR ,  2019 )  OR  LIMIT-TO ( PUBYEAR ,  2018 )  OR  LIMIT-TO ( PUBYEAR ,  2017 )  OR  LIMIT-TO ( PUBYEAR ,  2016 )  OR  LIMIT-TO ( PUBYEAR ,  2015 )  OR  LIMIT-TO ( PUBYEAR ,  2014 )  OR  LIMIT-TO ( PUBYEAR ,  2013 )  OR  LIMIT-TO ( PUBYEAR ,  2012 )  OR  LIMIT-TO ( PUBYEAR ,  2011 ) )

**Search:** #1 AND #2 AND #3 AND #4
