# Supplementary material for: Public Health Surveillance Systems in the Eastern Mediterranean Region: Bibliometric Analysis of Scientific Literature
Source: JMIR Public Health Surveill. 2021 Nov 1;7(11):e32639. doi: 10.2196/32639 (PMC8593796; doi:10.2196/32639)
Supplement: Multimedia Appendix 2 [file publichealth_v7i11e32639_app2.docx]

**Appendix 2**


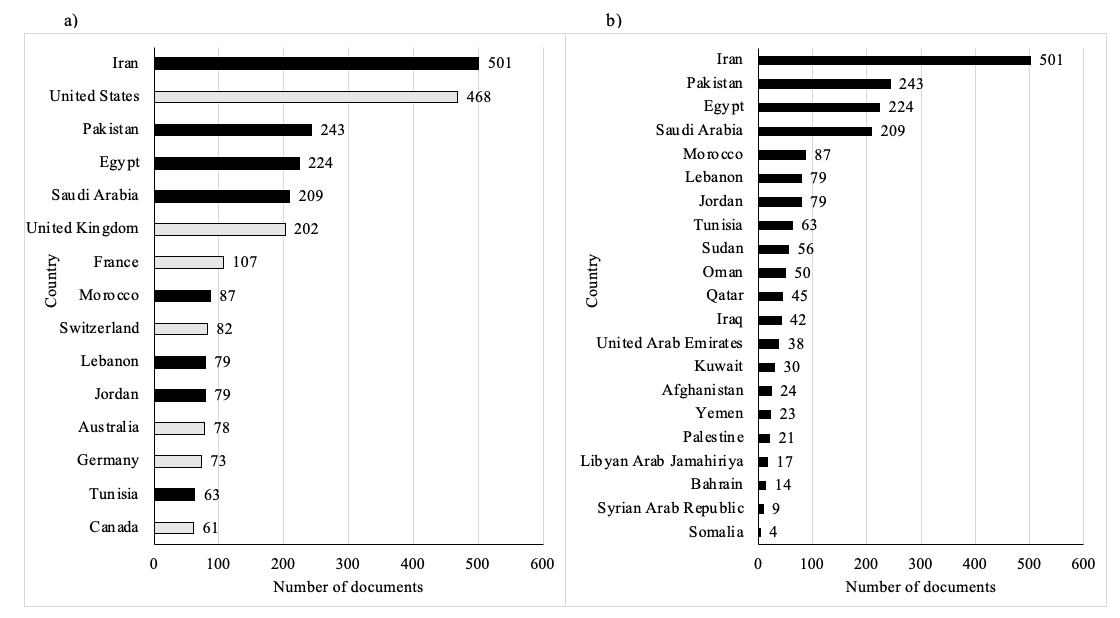


**Figure S1.** Number of published documents in the field of public health surveillance in the Eastern Mediterranean Region (EMR), between 2011 and 2021, (a) by country worldwide and (b) by countries in the EMR.


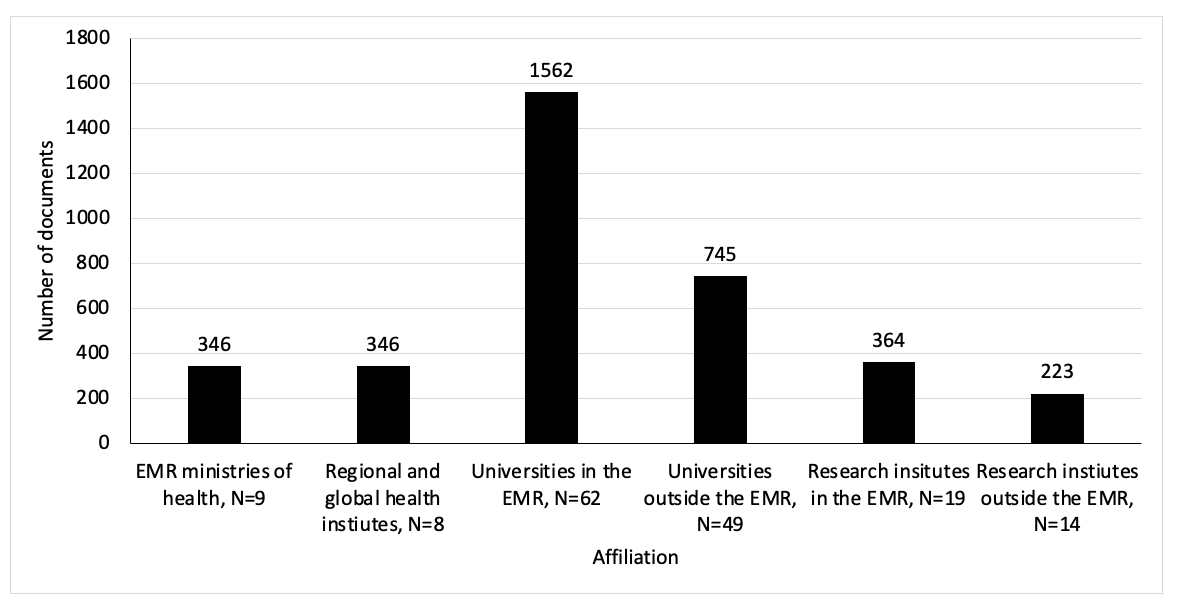


**Figure S2.** Affiliations of the published literature in the field of public health surveillance in the Eastern Mediterranean Region between 2011 and 2021. EMR: Eastern Mediterranean Region
